# Brain resting‐state networks in adolescents with high‐functioning autism: Analysis of spatial connectivity and temporal neurodynamics

**DOI:** 10.1002/brb3.878

**Published:** 2018-01-17

**Authors:** Antoine Bernas, Evelien M. Barendse, Albert P. Aldenkamp, Walter H. Backes, Paul A. M. Hofman, Marc P. H. Hendriks, Roy P. C. Kessels, Frans M. J. Willems, Peter H. N. de With, Svitlana Zinger, Jacobus F. A. Jansen

**Affiliations:** ^1^ Department of Electrical Engineering Eindhoven University of Technology Eindhoven The Netherlands; ^2^ Department of Neurology Maastricht University Medical Center Maastricht The Netherlands; ^3^ Department of Behavioral Sciences Epilepsy Center Kempenhaeghe Heeze The Netherlands; ^4^ Donders Institute for Brain, Cognition and Behaviour Radboud University Nijmegen Nijmegen The Netherlands; ^5^ School for Mental Health and Neuroscience Maastricht University Medical Center Maastricht The Netherlands; ^6^ Department of Radiology Maastricht University Medical Center Maastricht The Netherlands; ^7^ Department of Medical Psychology Radboud University Nijmegen Medical Centre Nijmegen The Netherlands

**Keywords:** autism spectrum disorder, functional MRI, Granger causality, high‐functioning autism, independent component analysis, resting‐state brain connectivity, temporal neurodynamics

## Abstract

**Introduction:**

Autism spectrum disorder (ASD) is mainly characterized by functional and communication impairments as well as restrictive and repetitive behavior. The leading hypothesis for the neural basis of autism postulates globally abnormal brain connectivity, which can be assessed using functional magnetic resonance imaging (fMRI). Even in the absence of a task, the brain exhibits a high degree of functional connectivity, known as intrinsic, or resting‐state, connectivity. Global default connectivity in individuals with autism versus controls is not well characterized, especially for a high‐functioning young population. The aim of this study is to test whether high‐functioning adolescents with ASD (HFA) have an abnormal resting‐state functional connectivity.

**Materials and Methods:**

We performed spatial and temporal analyses on resting‐state networks (RSNs) in 13 HFA adolescents and 13 IQ‐ and age‐matched controls. For the spatial analysis, we used probabilistic independent component analysis (ICA) and a permutation statistical method to reveal the RSN differences between the groups. For the temporal analysis, we applied Granger causality to find differences in temporal neurodynamics.

**Results:**

Controls and HFA display very similar patterns and strengths of resting‐state connectivity. We do not find any significant differences between HFA adolescents and controls in the spatial resting‐state connectivity. However, in the temporal dynamics of this connectivity, we did find differences in the causal effect properties of RSNs originating in temporal and prefrontal cortices.

**Conclusion:**

The results show a difference between HFA and controls in the temporal neurodynamics from the ventral attention network to the salience‐executive network: a pathway involving cognitive, executive, and emotion‐related cortices. We hypothesized that this weaker dynamic pathway is due to a subtle trigger challenging the cognitive state prior to the resting state.

## 1 INTRODUCTION

Autism spectrum disorder (ASD) is a heterogeneous neurodevelopmental disorder, which is characterized by persistent deficits in social communication and social interaction across multiple contexts and restricted, repetitive patterns of behavior, interest, or activities (*DSM–V*). Although not part of the diagnostic classification and not formal subcategories of ASD, a distinction is also often made between low‐functioning autism (LFA) and high‐functioning autism (HFA). No consensus criteria regarding LFA and HFA exist, but high‐functioning individuals with autism tend to have a “normal” IQ (Barendse et al., 2013). Recently, increasing interest has been focused on abnormalities in (functional)organization of specific brain regions, or networks, related to cognitive functions such as working memory, executive function, visual attention, and language processing (Anderson et al., 2011; Cherkassky, Kana, Keller, & Just, 2006). Many task‐based fMRI studies report that ASD is associated with either weaker or stronger connectivity between various structures (Monk, Peltier, Wiggins, & Weng, 2009). However, focusing on the “resting‐state” (i.e., task free) in fMRI provides a different domain to measure cortical synchronization patterns. Indeed, in the past decade, functional connectivity of resting‐state fMRI data is rapidly emerging as a highly efficient and powerful tool for in vivo mapping of neural circuitry in the human brain (Zuo et al., 2010). Thus far, resting‐state functional connectivity MRI studies in autism provide inconsistent results, that is, showing either under‐ or hyperconnectivity in similar investigated brain regions (Monk et al., 2009; Müller et al., 2011; Rane et al., 2015; Uddin, Supekar, Menon, Hutchison, & Williams, 2013). But those studies mainly assessed within‐network connectivity, that is, between hubs/ROIs connectivity. And evidence shows that for adolescents and adults, the impaired connectivity is to be found between, rather than within, large‐scale networks (Bos et al., 2014; Nomi & Uddin, 2015; Redcay et al., 2013; Tyszka, Kennedy, Paul, & Adolphs, 2014). To extract those large‐scale resting‐state networks and their associated time series, neuroimaging researchers have adopted a multivariate signal processing method known as independent component analysis (ICA). This data‐driven method needs no a priori on the measured signals, and hence, really suitable for resting‐state analysis (Beckmann, DeLuca, Devlin, & Smith, 2005; Thomas, Harshman, & Menon, 2002). Although ICA can provide spatial and temporal information about anatomical regions that show similar functional connectivity, it does not reveal causal relationships between components, that is, the effectiveness—directionality and strength—of the connectivity (Deshpande, LaConte, James, Peltier, & Hu, 2009; Liao et al., 2010). And recent evidence suggests that not the topology (structural and functional maps), but rather the dynamics of the network can better describe the disorder (Chen, Cai, Ryali, Supekar, & Menon, 2016; Deshpande, Libero, Sreenivasan, Deshpande, & Kana, 2013; Hanson, Hanson, Ramsey, & Glymour, 2013; Kana, Uddin, Kenet, Chugani, & Müller, 2014; Wicker et al., 2008). Hence, we also extract causality measures, which we call “temporal neurodynamics” in this paper, to represent temporal causal effect dependencies between RSNs. Temporal neurodynamics can be visualized using the Wiener–Granger causality test (Bressler & Seth, 2011; Granger, 1969) and its derived causality magnitude *F* upon two brain signals (time series). Here, the time series represent the RSN low‐frequency oscillations, extracted from ICA. Therefore, in this study, we focus on large‐scale networks, their shape and strength (within‐network spatial connectivity), and their effective connectivity with other large‐scale networks (between‐network neurodynamics). Regarding the potentially impaired networks, we also focus only on networks involving saliency, executive function, ventral attention network, and the default mode network, as those well‐known networks have shown atypical connectivity within and between networks (Anderson, Ferguson, & Nielsen, 2013; Keown et al., 2017; Nomi & Uddin, 2015).

Finally, we also test, using two resting‐state scans (rs‐scan 1 and 2) and a 1‐back visual task‐based fMRI in‐between, the hypothesis that a task‐based fMRI scan prior to a resting‐state scan session influences the post‐task resting‐state connectivity (Barttfeld et al., 2012; Hassan Saleh, 2011). Indeed we could expect a change in neurodynamics, and brain flexibility, after a cognitively demanding task in ASD, while controls would recover faster and should show none or less significant between‐resting‐state scans changes. Therefore, not only between‐group difference is analyzed, but also the between‐scan (recovery) effects (within the groups).

Differences between HFA and controls may be present in the composition of the spatial network organization (connectivity) and/or in the temporal neurodynamics (causal effect).

## 2 MATERIALS AND METHODS

### 2.1 Participants

Thirteen adolescents with ASD and 13 age‐ and IQ‐matched controls participated in this study. All participants were between 12 and 18 years old. Individuals with ASD were recruited from De Berkenschutse, a special secondary education school in Heeze (the Netherlands). All adolescents in the control group were recruited through an advertisement in a (local) newspaper and visited regular secondary schools in various regions of the Netherlands. Written informed consent was also obtained from the next of kin, caretakers, or guardians on behalf of the adolescents enrolled in this study. Inclusion criteria for the adolescents with HFA were established diagnostic criteria according to the *DSM–IV*, as well as the autism algorithm cut‐offs on the Autism Diagnostic Observation Schedule (ADOS) (Barendse et al., 2013; de Bildt et al., 2009). Inclusion criterion for the control group was no history of psychiatric illness. Adolescents in the control group were excluded if they and/or one of their siblings and/or parent(s) had a diagnosis of ASD. Further exclusion criteria for both groups were a comorbid psychiatric disorder, a significant hearing or visual impairment, an inability to speak/understand the Dutch language, and/or a comorbid central neurologic or other somatic disorder.

Table 1 shows the means and standard deviations (SD) of the ages in months and the Wechsler scores: the verbal comprehension index (VCI), perceptual organization index (POI), freedom from distractibility index (FDI), and full‐scale intelligence quotient (FSIQ). Using the analysis of variance (ANOVA) statistical method, we assessed the differences in the conditions (intelligence scores) of both groups.

**Table 1:** Demographic and descriptive data of ASD and control adolescents

Measure	ASD*M* (*SD*)	Controls*M* (*SD*)	Difference*F*(1, 24)	*p* b
Gender	12 male, 1 female	12 male, 1 female	—	—
Age (years)	15.3 (1.2)	14.5 (1.3)	2.89	.102
Verbal comprehension index	117.1 (9.0)	117 (10.4)	0.00	.968
Perceptual organization index	114.0 (5.8)	109.1 (7.8)	4.85	.038
Freedom from distractibility index	99.5 (14.5)	101.9 (14.6)	0.19	.670
Full‐scale IQ	116.7 (5.0)	113.2 (7.8)	1.92	.179
Autism Diagnostic Observation Schedule^a^ (number of patients)	2 (6)1 (5)0 (2)	0 (13)	—	—
Framewise displacement (mm)				
Scan 1	0.089 (0.043)	0.092 (0.034)	0.063	.80
Scan 2	0.082 (0.034)	0.079 (0.033)	0.033	.86

a 2 = autistic disorder, 1 = ASD, and 0 = no diagnosis according to ADOS.

b A *p* *< *0.05 means that a score or a characteristic (rows of the table) differs significantly between the two cohorts.

The study protocol was approved by the Medical Ethical Commission of the Maastricht University Medical Center.

### 2.2 Image acquisitions

MRI was performed on a 3.0‐Tesla unit (Philips Achieva) equipped with an 8‐channel receiver‐only head coil. For anatomical reference, a T1‐weighted 3D fast (spoiled) gradient echo sequence was acquired with the following parameters: repetition time (TR) 8.2 ms, echo time (TE) 3.7 ms, inversion time (TI) 1,022 ms, flip angle 8°, voxel size 1 × 1 × 1 mm^3^, field of view (FOV) 240 × 240 mm^2^, 150 transverse slices. Then, resting‐state fMRI data were acquired using the whole‐brain single‐shot multislice BOLD echoplanar imaging (EPI) sequence, with TR 2 s, TE 35 ms, flip angle 90°, voxel size 2 × 2 × 4 mm^3^, matrix 128 × 128, 32 contiguous transverse slices per volume, and 210 volumes per acquisition; resulting in total resting‐state acquisition of 7 min.

The resting‐state scans were performed twice with an 8‐min lasting 1‐back test for working memory assessment in‐between. This 1‐back test was performed to assess the working memory processes. For this memory task, pictures of houses or faces (neutral and smiling faces) were displayed randomly at regular intervals. Then, patients and controls were asked to indicate when the current stimulus (pictures) matched the previous picture (Koshino et al., 2008); for more details on the 1‐back task, see Supporting Information SI2. For both resting‐state scans, participants were instructed to lie with their eyes closed, and to think of nothing but not to fall asleep.

### 2.3 Data preprocessing

Data analysis was carried out using FMRIB Software Library (FSL; www.fmrib.ox.ac.uk/fsl). The following preprocessing was applied (van der Kruijs et al., 2014): discard of the first 3 volumes (=6 s) allowing the magnetization to reach equilibrium; rigid‐body motion correction (Jenkinson, Bannister, Brady, & Smith, 2002); nonbrain tissue removal; slice‐timing correction; registration to the Montreal Neurological Institute (MNI) standard space (2 mm isotropic); spatial smoothing using a Gaussian kernel of 4.0 mm full width at half‐maximum (FWHM); grand‐mean intensity normalization; and high‐pass temporal filtering at 100 s (0.01 Hz). After these preprocessing steps, one autistic subject and the second scan of a control participant were rejected because of a too large head motion: absolute displacement (mean) *>*1 mm with a maximal relative displacement (between two consecutive slice) *>*3 mm. Also, we reported the average framewise displacement (FD) in mm for each group and rs‐scan in Table 1.

### 2.4 Group independent component analysis

A single group‐level ICA was performed across all subjects and all scans from both HFA and control groups using probabilistic ICA as implemented in FSL multivariate exploratory linear optimized decomposition into independent components (MELODIC). First, the previously preprocessed 4D dataset was temporally transformed by concatenation into a single time series. This new 4D image was, then, separated into 34 independent components (ICs). The number of components was arbitrarily set to 34 as it seems to be a good trade‐off to get a sufficient number of relevant networks (around ten), without splitting them into subcomponents (Wang et al., 2011). To obtain the components, group probabilistic ICA processing steps were applied to the temporally concatenated 4D image: masking out nonbrain voxels, voxel‐wise demeaning of the data, and normalization of the voxel‐wise variance. Subsequently, the preprocessed data were projected into a 34‐dimensional subspace using probabilistic principal component analysis. Then these observations were decomposed into sets of vectors which describe signal variations across the temporal domain (time courses), the session/subject domain, and the spatial domain (maps) by optimizing for non‐Gaussian spatial source distributions using a fixed‐point iteration technique (Hyvärinen, 1999). The resulting estimated component maps were divided by the standard deviation of the residual noise and threshold at a posteriori probability threshold of *p > *.5 (i.e., an equal loss is placed on false positives and false negatives) by fitting a Gaussian/gamma mixture model to the histogram of intensity values (Beckmann et al., 2005).

### 2.5 Resting‐state networks selection

The most relevant group‐level IC maps (of 34) were selected according to the following three steps. First, group‐level IC maps with more than 33% of the estimated spectral power in high frequencies (*>*0.1 Hz) were excluded to keep only networks within the low‐frequency range of 0.1–0.01 Hz (Lowe, Mock, & Sorenson, 1998; Tyszka et al., 2014). Second, Smith et al. (2009) described the major covarying networks in the resting brain and created a template of these RSNs widely used in resting‐state fMRI studies. With this template and our remaining group maps, a function, using the “goodness‐of‐fit” approach was created and applied (Greicius, Srivastava, Reiss, & Menon, 2004; Vanhaudenhuyse et al., 2010). Finally, the third step consisted in a visual inspection of each component spatial profile to verify the consistency and ensure the effectiveness of the two previous steps. Plus, this last step allows us to select other known and well‐described networks that are not in Smith and colleagues’ template, but still comply with first selection step.

### 2.6 Spatial RSN analysis between groups

The first level of the voxel‐wise group analysis was performed using dual‐regression (Beckmann, Mackay, Filippini, & Smith, 2009). The aim of this process is to obtain, from the group IC maps, subject‐specific IC maps. Dual‐regression involves two general linear models (GLM). First, the group IC maps were used as spatial regressors against the preprocessed individual fMRI scans. This results in single‐subject time courses for each component separately. Then, these time courses were normalized to unit variance to test both the “shape” and “amplitude” of the RSN. In the second GLM, these normalized individual time courses were used as temporal regressors against the preprocessed individual fMRI images, leading to subject‐specific IC maps for each subject's scan. As there were two subject‐specific spatial maps per IC for each individuals (one per scan), before running final group‐level analysis, we merged and averaged these two IC maps per subject. We also compared the two groups for each scan separately, that is, without the merging and averaging of the RSN maps, as explained below.

The second level of the group analysis consisted in getting the effects of within‐group means (control group average *>*0; HFA group average*>*0) and between‐group differences (HFA *> *control; control *> *HFA). This was assessed using nonparametric permutation testing (5000 permutations), with FSL's randomize tool (Nichols & Holmes, 2002). For each RSN, the resulting statistical maps were threshold at *p < *.05, family‐wise error (FWE) corrected with the threshold‐free cluster enhancer (TFCE) technique (Smith & Nichols, 2009). Finally, nuisance regressors describing age, IQ, and relative gray matter volume were added to the model in a second experiment, to observe their possible effects on the between‐group contrast maps.

### 2.7 Temporal dynamics of RSNs

The statistical Granger causality (G‐causality) allows us to assess causality among two signals. One signal Y is said to Granger cause another signal X, if the past of Y and X can better predict the future of X rather than with the past of X only (Zaremba & Aste, 2014). In this study, we use this principle to evaluate pairwise multivariate conditional Granger causalities of our independent components (resting‐state networks). The assessment is performed on each pair of subject‐specific RSNs time series and repeated for each resting‐state scan, using the multivariate Granger causality (MVGC) toolbox (Barnett & Seth, 2014). This gives us estimates *F* of Granger causality magnitudes for each network pairs, subject, and scan. Furthermore, with two‐sample two‐tailed *t*‐tests we compare these G‐causality magnitudes between the two groups (HFA vs. controls) to determine different patterns of neuronal dynamics of the resting state (effective connectivity). We also perform this test for the two resting‐state scan sessions to assess whether or not a previous task‐based fMRI scan can trigger and/or change the dynamics of resting‐state connectivity, that is, the causality between RSNs. Finally, we assess if these changes differ between patients and controls. According to several studies, people with ASD show differences mainly in frontal and temporal cortices, default mode parts, and also within networks related to social interaction (Anderson et al., 2013; Hanson et al., 2013; Keown et al., 2017; Nomi & Uddin, 2015). Therefore, we selected the RSNs located mainly in frontotemporal cortices and/or consisting of sociocognitive brain parts. These networks are assessed and compared with the method described above.

## 3 RESULTS

For each group, age, gender, intelligence scores, ADOS diagnostic score, and mean framewise displacement (in mm) per group and per scan are displayed in Table 1. Only the perceptual organization index (POI) score showed a significant difference between the adolescents with ASD and the controls (*p < *.05, Table 1).

### 3.1 RSN selection and between‐groups spatial analysis

**Figure 1:**
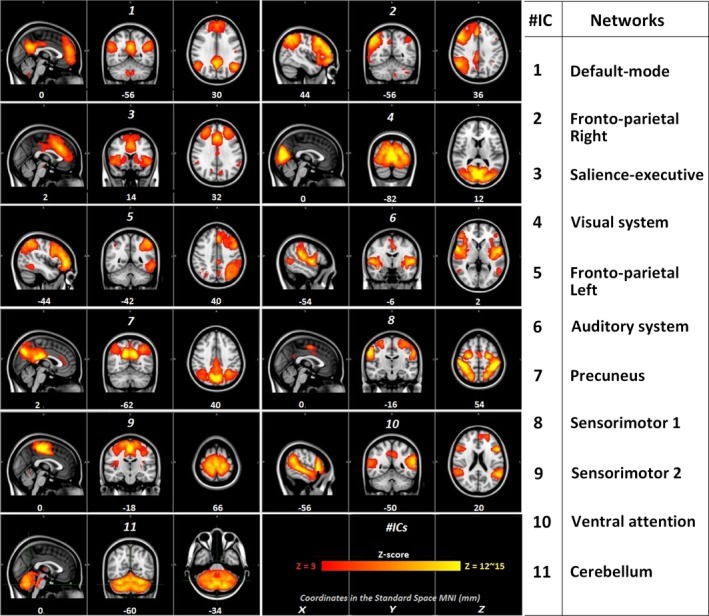
Relevant components extracted from the group‐level ICA. Relevant components extracted from the 34 group IC maps overlaid in color on the MNI standard brain (2 × 2 × 2 mm). Names of the networks are in the right‐side table. The colorbar is threshold between 3 and 15 (*z*‐score). MNI coordinates are in mm. The left hemisphere corresponds to the right side in the images (radiological convention)

We extracted functionally relevant group ICs based on the resting‐state template from Smith et al. (2009). After visual inspection, we also included the widely described ventral attention network (Corbetta, Patel, & Shulman, 2008; Farrant & Uddin, 2015; Fox, Corbetta, Snyder, Vincent, & Raichle, 2006). Finally for further analysis, we described our executive control network as the salience‐executive network, since it involves, not only prefrontal and posterior cingulate cortices (for executive function), but also the salient network compounded with the anterior insular and anterior cingulate cortices (Menon & Uddin, 2010; Sala‐Llonch, Bartrés‐Faz, & Junqué, 2015). The 11 networks that we finally obtained are depicted in Figure 1. Those RSNs were found in both HFA and controls by testing the subject‐specific maps of these networks (after the dual regression). In those relevant networks, the group effects (group mean *> *0) are present and strongly consistent with the whole‐group (ASD + Controls) networks (see Figure S1). Also, after a nonparametric permutation test (5,000 permutations) threshold at *p < *.05, TFCE corrected for FEW, no voxels, in any components, were significant in the second‐level group analysis for the ASD *> *control and control *> *ASD contrasts. The same results occurred when comparing the groups for each scan separately. In the second equivalent statistical analysis, where age, IQ level, and gray matter density were added as covariates, again, no voxels in any component survived at the same threshold (*p* *<* .05, FWE corrected; see Table S1 for more details). Hence, statistically, the strength and the extent of each network (functional connectivity) were similar in both groups.

### 3.2 Temporal dynamics of the RSNs

The causal analysis to detect temporal dynamics differences was made upon the four most relevant RSNs that involved frontoparietal and temporal cortices, and networks related to social cognition (Anderson et al., 2013; Hanson et al., 2013; Keown et al., 2017; Nomi & Uddin, 2015). Therefore, we selected the default mode network (IC 1, Figure 1), the salience‐executive system (IC 3), the ventral attentional network (IC 10), and the auditory system (IC 6). Pairwise conditional Granger causality magnitudes, in average, within each group for the first and second resting‐state scan sessions were significant (*p* < .05 FDR corrected). This was found for the four aforementioned selected prefrontal and temporal RSNs and for both groups. Also, the positivity of the normality test (Kolmogorov–Smirnov) for the distribution of the causalities among each group allowed us to use the two‐sample two‐tailed *t*‐test to compare HFA adolescents’ G‐causalities with those of the control group.

**Figure 2:**
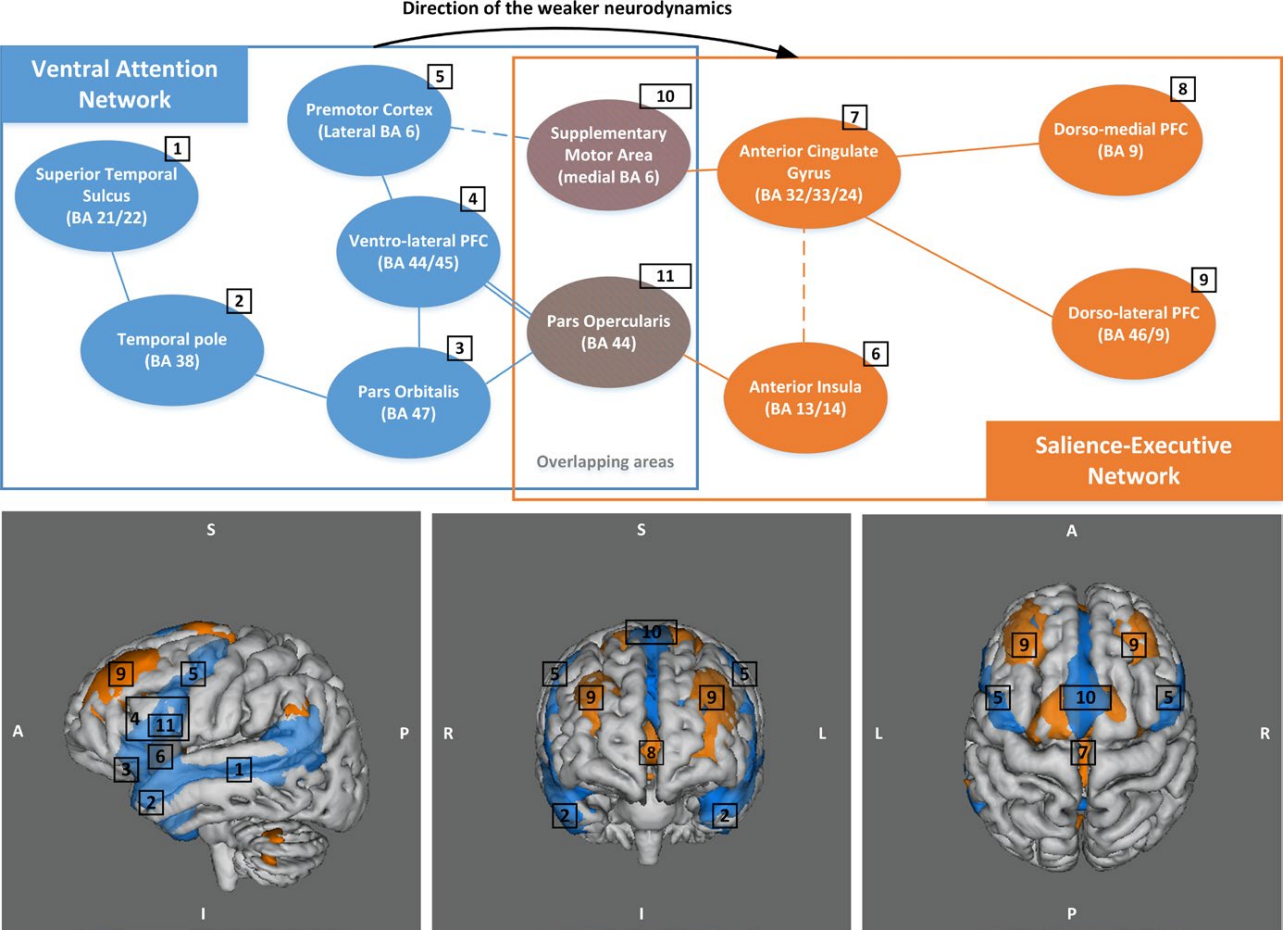
Visualization of the weaker neurodynamic pattern in adolescent with HFA (in the post‐task resting state). Visualization of the two RSNs, salience executive in blue and ventral attention in orange, where the causal dynamics is weaker in HFA for the second (post‐task) resting‐state scan, in the direction from ventral attention to salience executive. The scheme above shows the different cortices involved in these two networks and the overlapping areas. Solid lines and dashed lines describe direct cortico‐cortical physical link and indirect connections (through white matter and/or basal ganglia), respectively. The pars opercularis is a part of the ventrolateral PFC which is shown with the double line. On the bottom part, anatomical visualization of the two abovementioned RSNs (from group IC contrast maps) are displayed (threshold at *z* > 2.6, i.e., *p* < .01). BA, Brodmann area; PFC, prefrontal cortex

In the first resting‐state scan, none of the pairwise causalities differed significantly between ASD and controls. We also found no significant differences in causality within the control group, that is, when comparing first rs‐scan and second rs‐scan. However, dynamic RSN patterns did differ within the ASD cohort (first rs‐scan vs. second rs‐scan) and significantly diverged from control adolescents only in the second resting‐state scan. The latter result shows a significant lower value of Granger causality between the ventral attention and salience‐executive networks in the ASD group as compared with control: mean F (Granger causality value) for ASD = 0.028 (*SD* = 0.015); mean F controls = 0.058 (*SD* = 0.031); *t*(24)  = 3.17, *p*‐value = .0042. Figure 2 shows this directed causal connection and displays in more detail the cortical regions involved in these two networks.

## 4 DISCUSSION

In the present study of high‐functioning adolescents with autism, resting‐state whole‐brain functional connectivity was examined. No evidence was found for any significant difference in brain spatial connectivity between the two populations. However, our results did show that patterns in temporal neurodynamics, that is, causal effects of one RSN on another, differ between the groups. In contrast with controls, HFA display a significant difference in temporal neurodynamics between resting‐state fMRI sessions 1 and 2. Furthermore, in contrast with the first resting‐state scan, temporal neurodynamics differ significantly between HFA and controls during the second resting‐state session. The primary findings of similar functional connectivity between the two cohorts challenge the theory that the autistic brain is globally underconnected (Belmonte, 2004; Uddin, Supekar, Menon, et al., 2013). This can be explained by differences in scan protocols, in postprocessing methods (ICA vs. seed based) and mainly because of the population (type of the ASD, number, and ages). However, our findings of similar functional resting‐state network, that is, similar within‐network functional connectivity are corroborated by other studies on adolescents and adults with high‐functioning autism (Bos et al., 2014; Tyszka et al., 2014; Uddin, Supekar, Menon, et al., 2013). More recently, Nomi and Uddin (2015) also showed that adolescents with ASD do not have altered within‐network functional connectivity. But interestingly, by means of correlation between the RSNs time series, they obtained evidence of impaired between‐network connectivity in the adolescents with autism. The pairwise temporal correlations used in their study can be seen as (undirected) instantaneous causality. Hence, their results of between‐network hypoconnectivity in ASD population is partially (only instantaneous causality) in line with our results of weaker neurodynamics in autism, which are discussed in the next paragraph.

To go further and detect strength and directionality in causality between RSNs, we used Granger causality upon four relevant sociocognitive RSN time series. The two RSNs showing differences in effective directed connectivity (neurodynamics) are the ventral attention network and the salience‐executive control network. The ventral attention contains mainly the left and right superior temporal sulci (STS), the temporal poles, the ventrolateral and orbital cortices, and lateral premotor cortex. This pathway is known to code for visual recognition and identification, and for emotional processes. The temporal pole is known to play a role in functions that tend to be weak in autism: social and emotional processing, including face recognition and the theory of mind (Kana et al., 2015; Olson, Plotzker, & Ezzyat, 2007). Also, the STS has been postulated to be a critical component of the abnormal neural circuitry underlying deficits in social perception in autism (Redcay, 2008). The STS projects information toward prefrontal cortices (mainly the medial and lateral) which are part of the salience‐executive network. This salience‐executive control network involves the anterior cingulate gyrus (ACC), the anterior insular cortex (AI) as well as the dorsomedial and dorsolateral prefrontal cortices, and the supplementary motor area (SMA). These ROIs are involved in cognitive processes such as working memory, reasoning, task flexibility, problem solving, planning, and execution (Chan, Shum, Toulopoulou, & Chen, 2008). The AI cortex is a brain structure implicated in disparate cognitive, affective, and regulatory functions, including interoceptive awareness, emotional responses, and empathic processes (Menon & Uddin, 2010). More specifically, Dapretto et al. (2006) propose a mirror neuron system (MNS) dysfunction in children with ASD (Dapretto et al., 2006). Notably, they affirm that the MNS activity in the pars opercularis is consistently present during imitation, action observation, and intention understanding; and this pars opercularis combined with the insula and limbic activity (e.g., in the ACC) may mediate the understanding of others’ emotional states. However, the absence of mirror neuron activity in the frontal part of the MSN (pars opercularis) leads this emotional process to be weaker in ASD. This weakened “theory‐of‐mind” network has been further confirmed in children and adolescents (Kana et al., 2015).

The association AI/ACC, also termed the salient network, plays a role in dynamic switching between brain networks in reaction to cognitively demanding tasks (switch default mode network/executive network; Menon & Uddin, 2010; Sridharan, Levitin, & Menon, 2008). A review study reports that this critical system (salience network) is impaired in ASD, and that the AI region has demonstrated hypoactivity in individuals with ASD across a wide variety of social cognitive task paradigms (Di Martino, Ross, et al., 2009; Uddin & Menon, 2009). All these findings illustrate that activations in brain areas implicated in the ventral attention and salience‐executive RSNs are known to be weaker in the ASD population. Also, areas in the salient network and the MNS, that is, the causal flow “bridge” area (Figure 2), are critical during self‐ and other‐related social and affective processes, and also known to be underactivated in ASD (Barttfeld et al., 2012; Kana et al., 2015). In line with the previously mentioned studies conducted with the help of socioemotional cognitive task‐based fMRI, we observed the same weaknesses in whole‐brain resting‐state functional connectivity, but only when analyzing temporal dynamics. The extracted RSNs do not have the same pattern of temporal dynamics, that is, the influence of one RSN on another varies between the two cohorts: the causal connectivity between the salience‐executive and ventral attention networks (in the direction of ventral attention *→* salience‐executive) is significantly weaker in the HFA population, but only in their post‐task resting state. This impaired temporal neurodynamics suggests failing bridging of the emotional states regulated in the ventral attentional to the decision‐making‐oriented salience‐executive control system. This may therefore be described as a more rigid system in terms of the emotional‐executive bridge, which can be seen as an endogenous to exogenous (self to other) dynamic process failure as suggest by the literature (Di Martino, Shehzad, et al., 2009; Ebisch et al., 2011; Menon & Uddin, 2010; Uddin, Supekar, Ryali, & Menon, 2011; Uddin, Supekar, Lynch, et al., 2013). Finally, in our study, the differences in temporal neurodynamics were only found in the second resting‐state session. An 1‐back working memory task‐based scan was performed in between the two resting‐state scan sessions. We therefore hypothesize that abnormal temporal neurodynamic patterns in HFA were triggered by the working memory task, involving not only working memory, but also attentional and emotional (in terms of face and emotion recognition) processes. This could be explained by a reduced cognitive flexibility (or more rigidity) in the post‐task resting‐state connectivity in ASD, reducing the between‐networks dynamics, showing a more brain state dependency of connectivity pattern in autism compared to controls, as shown recently in literature (Barttfeld et al., 2012; Chen et al., 2016; Douw, Wakeman, Tanaka, Liu, & Stufflebeam, 2016; Uddin et al., 2015).

### 4.1 Limitations

One of the main challenges in applying G‐causality upon fMRI BOLD signals is the problem of the hemodynamic response function (HRF) changes. Inter‐regional HRF variation has been argued to affect G‐causality analysis (David et al., 2008). But the Granger causality method implemented in MVGC software, used in our study, has been proven to be robust to changes in HRF properties (Seth, Chorley, & Barnett, 2013). A second limitation with our technique is the relatively long sample intervals (TR) of classic fMRI protocols (usually ranging from 1 s to 3 s). Indeed, our TR of 2 s is substantially longer than typical interneuron delays. However, since we examine changes (of differences) in G‐causality rather than attempting to find a ground truth G‐causality pattern that limitation is not significant (Barnett & Seth, 2014).

Finally, even though our statistical analyses are properly controlled for multiple comparisons and for type I error (false positive), cautious interpretation of the results is in order. Especially misses (type II error, or false negative) could have occurred for the results of similar spatial network connectivity (miss of spatial differences).

### 4.2 Methodological recommendations

For future application, we state that neurodynamics provide alternative strategies when ICA analysis does not yield differences for a cross‐sectional analysis. Also, conversely, where ICA does show differences in functional connectivity between two populations (or more), we advise not to use Granger causality analysis on temporal trends of ICs, but rather on raw ROI signals (with same ROI location for both groups). Finally, our findings show that tasks prior to resting‐state acquisition scan can have an effect on the results of an effective connectivity analysis.

## 5 CONCLUSION

We find no significant differences in resting‐state brain connectivity between high‐functioning adolescents with ASD and the control group at the whole‐ brain level. However, the extracted RSNs do not have the same pattern of temporal dynamics, that is, the influence of one RSN on another is different between the two cohorts. In particular, the causal connectivity between the salience‐executive and the ventral attention networks (in the direction of ventral attention → salience‐executive) is significantly weaker in the HFA population in the second resting‐state scan, after challenging sensitive functions for HFA adolescents. These two networks link cortices coding for face/object recognition and emotional processing with cortices of executive cognitive functions (attention, control, working memory, behavior). We hypothesize that changes in neurodynamics at rest in HFA are subtly triggered by challenging the cognitive state prior to the resting state. And these changes seem to appear in the dynamic connectivity between the networks functionally related to the previous cognitive task.

### Notes

Sample sizes for the groups have been calculated using previous fMRI studies on ASD with significant results of lower functional connectivity in working memory network, and ToM network in the autism cohorts (Kana et al., 2015; Koshino et al., 2008). Those studies had 13 TDC/13 ASD and 11 TDC/11 ASD, respectively. Post hoc power analyses using the significant results from the two papers lead to a power of 90% and 83%, respectively. Hence, using 13/13 patients/controls is sufficient and powerful enough for finding similar effect sizes.

Inclusion criteria were predetermined. For further analysis (such as group comparison) we had post hoc exclusion criteria such as a too large framewise displacement and/or bad registration to the standard brain (quality check after preprocessing the data).

Data were not anonymized and no blinding was performed during the analysis.

Finally, no informed consent for personal data sharing has been collected in this study. Therefore, the fMRI data are not publicly available.

## CONFLICT OF INTEREST

None declared.
