# Supplementary material for: Brain resting‐state networks in adolescents with high‐functioning autism: Analysis of spatial connectivity and temporal neurodynamics
Source: Brain Behav. 2018 Jan 17;8(2):e00878. doi: 10.1002/brb3.878 (PMC5822569; doi:10.1002/brb3.878)
Supplement: Supplementary file 1 [file BRB3-8-e00878-s001.pdf]

SI1 - Supporting Information

Multivariate Conditional Granger Causality theory

We use a multivariate Granger causality MATLAB toolbox to calculate the conditional granger causality magnitudes. Algorithms and functions of this toolbox are describe in Lionel Barnett's paper [1]. The following parts describe the mathematical theory and processes needed behind Granger causality. Geweke [2] describes the measure of the Granger causality as follows:

$$F_{\mathbf{Y}\to\mathbf{X}} \equiv \ln \frac{|\Sigma'_{xx}|}{|\Sigma_{xx}|} \tag{1}$$

In this equation, Σ'_{xx} is variance of the prediction error (residuals) of $\mathbf{X}_t$ excluding the possible causal influence of $\mathbf{Y}_t$, and Σ_{xx} is variance of the prediction error of $\mathbf{X}_t$ including the possible causal influence of $\mathbf{Y}_t$. To measure these variances, the vector autoregressive model (VAR) [3, 4, 5] is applied. This model is defined as

$$\mathbf{U}_t = \sum_{k=1}^{p} A_k \cdot \mathbf{U}_{t-k} + \boldsymbol{\varepsilon}_t \tag{2}$$

here, $\boldsymbol{U}_t$ is a time series, A_k - regression coefficients, ε - the residuals and p - the model order representing the number of past lags taken into account. This model order is calculated from time series using the Akaike Information Criterion (AIC) [6, 7]. If $\boldsymbol{U}_t$ is a multivariate vector composed if two time series $\boldsymbol{X}$ and $\boldsymbol{Y}$, the VAR model can be developed as shown in Eq. (4).

$$\mathbf{U}_t = \begin{pmatrix} \mathbf{X}_t \\ \mathbf{Y}_t \end{pmatrix} \tag{3}$$

$$\begin{pmatrix} \mathbf{X}_t \\ \mathbf{Y}_t \end{pmatrix} = \sum_{k=1}^{p} \begin{pmatrix} A_{xx,k} & A_{xy,k} \\ A_{yx,k} & A_{yy,k} \end{pmatrix} \begin{pmatrix} \mathbf{X}_{t-k} \\ \mathbf{Y}_{t-k} \end{pmatrix} + \begin{pmatrix} \boldsymbol{\varepsilon}_{x,t} \\ \boldsymbol{\varepsilon}_{y,t} \end{pmatrix} \tag{4}$$

with the covariance matrix of residuals expressed as

$$\Sigma \equiv cov \begin{pmatrix} \boldsymbol{\varepsilon}_{x,t} \\ \boldsymbol{\varepsilon}_{y,t} \end{pmatrix} = \begin{pmatrix} \Sigma_{xx} & \Sigma_{xy} \\ \Sigma_{yx} & \Sigma_{yy} \end{pmatrix} \tag{5}$$

By applying the Morf's variant of the Locally Weighted Regression (LWR) algorithm [8], which is very efficient for likelihood-based model selection such as AIC [9], we estimate the VAR parameters. If we consider the X-component of the regression (4), we obtain the following expression:

$$\mathbf{X}_t = \sum_{k=1}^{p} A_{xx,k} \cdot \mathbf{X}_{t-k} + \sum_{k=1}^{p} A_{xy,k} \cdot \mathbf{Y}_{t-k} + \boldsymbol{\varepsilon}_{x,t} \tag{6}$$

and if we suppose no conditional dependence between $\mathbf{X}$ and the past of $\mathbf{Y}$ ($A_{xy,1} = A_{xy,2} = ... = A_{xy,p} = 0$), we obtain the reduced regression:

$$\mathbf{X}_t = \sum_{k=1}^{p} A'_{xx,k} \cdot \mathbf{X}_{t-k} + \boldsymbol{\varepsilon}'_{x,t} \tag{7}$$

We finally get from (5) the following: $\Sigma_{xx} = cov(\boldsymbol{\varepsilon}_{x,t})$ and $\Sigma'_{xx} = cov(\boldsymbol{\varepsilon}'_{x,t})$.Hence, we get the Geweke measure of the Granger causality $F_{\mathbf{Y}\to\mathbf{X}}$ (1).

In our case, we have more than two time series, thus the Granger causality between one signal and another is depending on the information added by the past of the other signals. Let us now illustrate this conditional pairwise G-causality. Suppose we have

$$\mathbf{U}_t = \begin{pmatrix} \mathbf{X}_t \\ \mathbf{Y}_t \\ \mathbf{Z}_t \end{pmatrix}, \tag{8}$$

where $\mathbf{Z}_t$ is a third set of variables. We aim at eliminating any joint effect of $\mathbf{Z}$ on the inference of the G-causality $\mathbf{Y}$ to $\mathbf{X}$. Thus, analogously to (4), the full and reduced regression for the X-component becomes respectively

$$\mathbf{X}_t = \sum_{k=1}^{p} A_{xx,k} \cdot \mathbf{X}_{t-k} + \sum_{k=1}^{p} A_{xy,k} \cdot \mathbf{Y}_{t-k} + \sum_{k=1}^{p} A_{xz,k} \cdot \mathbf{Z}_{t-k} + \boldsymbol{\varepsilon}_{x,t} \tag{9}$$

and

$$\mathbf{X}_t = \sum_{k=1}^{p} A'_{xx,k} \cdot X_{t-k} + \sum_{k=1}^{p} A'_{xz,k} \cdot \mathbf{Z}_{t-k} + \boldsymbol{\varepsilon}'_{x,t} \tag{10}$$

which corresponds to (6) and (7) but with the inclusion of conditioning variables $\mathbf{Z}$ in both regressions. We can finally quantify the conditional G-causality as the log-likelihood ratio:

$$F_{\mathbf{Y}\to\mathbf{X}|\mathbf{Z}} \equiv \ln \frac{|\Sigma'_{xx}|}{|\Sigma_{xx}|} \tag{11}$$

which can be defined as "the degree to which the past of $\mathbf{Y}$ helps predict $\mathbf{X}$ over and above the degree of which $\mathbf{X}$ is already predicted by its own past *and the past of* $\mathbf{Z}$" [1].

Supporting information of no between-group difference in spatial RSN connectivity

Table S 1. FWE-corrected P-values of the first voxels (≥ 1) showing a between-group RSN spatial difference.

Network	p-value (corrected)			
	$Con > ASD$	$ASD > Con$	$Con > ASD$ adj*	$ASD > Con$ adj*
**Default-mode**	0.93	0.54	0.85	0.58
**Fronto-parietal R**	0.94	0.13	0.59	0.23
**Salience-executive**	0.39	0.36	0.65	0.66
**Visual system**	0.33	0.83	0.38	0.74
**Fronto-parietal L**	0.29	0.60	0.10	0.38
**Auditory system**	0.97	0.59	0.79	0.61
**Precuneus**	0.39	0.94	0.13	0.82
**Sensorimotor 1**	0.20	0.71	0.60	0.43
**Sensorimotor 2**	0.55	0.21	0.78	0.18
**Ventral attention**	0.12	0.90	0.26	0.96
**Cerebellum**	0.77	0.76	0.78	0.76

No voxels survived at the threshold $P = 0.05$. P-values are FWE-corrected for multiple comparison.

adj*: statistical maps adjusted for subjects' ages, IQ levels and gray matter volumes.

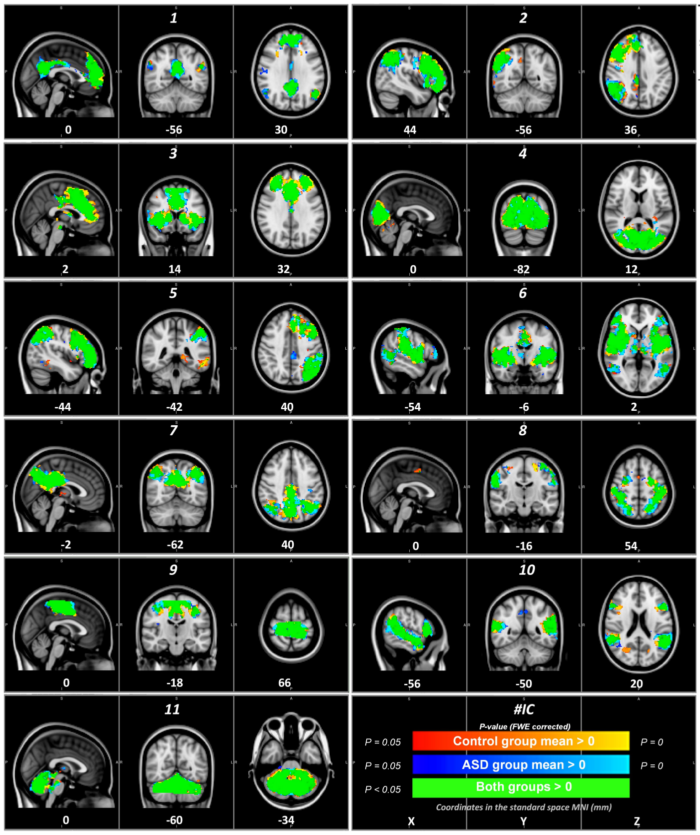

#IC	Network
**1**	Default-mode
**2**	Fronto-parietal Right
**3**	Salience-executive
**4**	Visual system
**5**	Fronto-parietal Left
**6**	Auditory system
**7**	Precuneus
**8**	Sensorimotor 1
**9**	Sensorimotor 2
**10**	Ventral attention
**11**	Cerebellum

Fig. S 1. Group mean effect per network. Red-yellow = Control group; Blue-lightblue = ASD group; Green = Overlapping of both groups; thresholded at $p < 0.05$ FWE corrected.

References

[1] Barnett L, Seth A. K, The mvgc multivariate granger causality toolbox: A new approach to granger-causal inference, J Neurosci Methods 223 (2014) 50–68.

[2] Geweke J. F, Measure of conditional linear dependence and feedback between time series, J. Am. Stat. Assoc. 79 (388) (1984) 907–915.

[3] Roebroeck A, Formisano E, Goebel R, Mapping directed influence over the brain using granger causality and fmri, NeuroImage 25 (1) (2005) 230–242.

[4] Zhou Z, Wang X, Klahr N. J, Liu W, Arias D, Liu H, et al., A conditional granger causality model approach for group analysis in functional magnetic resonance imaging, Magn Reson Imaging 29 (3) (2009) 418–433.

[5] Deshpande G, Santhanam P, Hu X, Instantaneous and causal connectivity in resting state brain networks derived from functional mri data, NeuroImage 54 (2) (2011) 1043–1052.

[6] Akaike H, A new look at statistical model identification, IEEE Trans. Autom. Control 19 (6) (1974) 716–723.

[7] Deshpande G, LaConte S, Jamas G. A, Peltier S, Hu X, Multivariate granger causality analyisis of fmri data, Hum. Brain Mapp 30 (4) (2009) 1361–1373.

[8] Morf M, Vieira A, Lee D. T. L, Keilath T, Recursive multichannel maximum entropy spectral estimation, IEEE Trans. Geosci. Electron 16 (2) (1978) 85–94.

[9] McQuarrie A. D. R, Tsai C.-L, Regression and time series model selection, World Scientific, Singapore, 1998.
