# Supplementary material for: Brain resting‐state networks in adolescents with high‐functioning autism: Analysis of spatial connectivity and temporal neurodynamics
Source: Brain Behav. 2018 Jan 17;8(2):e00878. doi: 10.1002/brb3.878 (PMC5822569; doi:10.1002/brb3.878)
Supplement: Supplementary file 2 [file BRB3-8-e00878-s002.pdf]

SI2 - Supporting information

1-back task fMRI protocol

The following procedure and parameters were used for the 1-back visual working memory task.

1-back Task description

The 1-back task looks at processing complex stimuli: social and non-social. The non-social complex stimuli are homes, the social complex stimuli are faces.

The participant should not remember one specific house / face, but consider whether two photos in a row are exactly the same. The participant should remember, not only the identity of the house / face, but also the specific feature (house: upper or lower adjusted / face: emotion angry, happy, fear).

Participant is instructed to press the "yes" button if the picture he/she are seeing is an exact copy of the previous one, and press the "no" button otherwise.

Here an example:

Sequentially (from left to right) the pictures are displayed as below. Red dots means what the participant should respond when he/she sees that very picture. The task alternates randomly between the houses and the faces (see next paragraph).

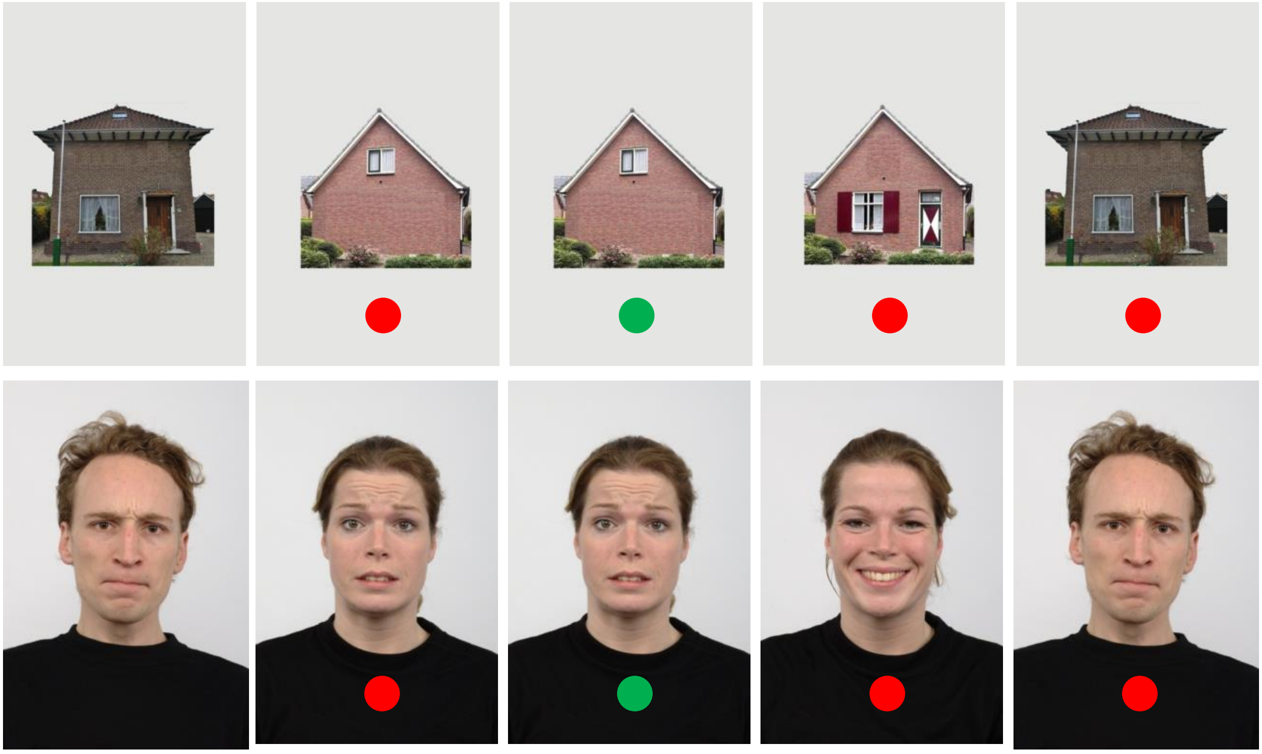

Task procedure

Before the participant goes into the scanner, the task will be practiced outside the scanner. The participant has already had extensive instructions (and practiced with the task) outside the scanner. The instructions in the scanner must therefore be limited to the literal instructions of the task as shown on the screen.

- During the anatomical scan, the participant can read the instructions on screen again.

- Before starting the fMRI scan: Read the instructions on the screen literally with the participant and see if he / she has any questions.

- Start the fMRI scan if the participant does not have any questions the fMRI scan. The task will then automatically start and run with the scan.

Note: If the participants wants feedback: Indicate that you cannot give it now because everything needs to be analyzed first and only if a group of people has been tested.

Task design

Below, the schedule for displaying the picture used for the 1-back visual task (1-minute period and at 50% task duty cycle).

8 min 1-back						1						1			
minuut	*1*	*minuut*	*2*	*minuut*	*3*	*minuut*	*4*	*minuut*	*5*	*minuut*	*6*	*minuut*	*7*	*minuut*	*8*
H28	1500 ms	F72	1500 ms	H37	1500 ms	H17	1500 ms	F41	1500 ms	H2	1500 ms	F69	1500 ms	F6	1500 ms
P0	500 ms	P0	500 ms	P0	500 ms	P0	500 ms	P0	500 ms	P0	500 ms	P0	500 ms	P0	500 ms
H30	1500 ms	F72	1500 ms	H40	1500 ms	H17	1500 ms	F64	1500 ms	H2	1500 ms	F14	1500 ms	F6	1500 ms
P0	500 ms	P0	500 ms	P0	500 ms	P0	500 ms	P0	500 ms	P0	500 ms	P0	500 ms	P0	500 ms
H30	1500 ms	F80	1500 ms	H40	1500 ms	H10	1500 ms	F3	1500 ms	H2	1500 ms	F15	1500 ms	F43	1500 ms
P0	500 ms	P0	500 ms	P0	500 ms	P0	500 ms	P0	500 ms	P0	500 ms	P0	500 ms	P0	500 ms
H30	1500 ms	F80	1500 ms	H1	1500 ms	H12	1500 ms	F2	1500 ms	H6	1500 ms	F15	1500 ms	F43	1500 ms
P0	500 ms	P0	500 ms	P0	500 ms	P0	500 ms	P0	500 ms	P0	500 ms	P0	500 ms	P0	500 ms
H43	1500 ms	F37	1500 ms	H3	1500 ms	H33	1500 ms	F2	1500 ms	H6	1500 ms	F40	1500 ms	F45	1500 ms
P0	500 ms	P0	500 ms	P0	500 ms	P0	500 ms	P0	500 ms	P0	500 ms	P0	500 ms	P0	500 ms
H43	1500 ms	F38	1500 ms	H9	1500 ms	H36	1500 ms	F2	1500 ms	H22	1500 ms	F17	1500 ms	F25	1500 ms
P0	500 ms	P0	500 ms	P0	500 ms	P0	500 ms	P0	500 ms	P0	500 ms	P0	500 ms	P0	500 ms
H25	1500 ms	F38	1500 ms	H41	1500 ms	H39	1500 ms	F9	1500 ms	H23	1500 ms	F17	1500 ms	F28	1500 ms
P0	500 ms	P0	500 ms	P0	500 ms	P0	500 ms	P0	500 ms	P0	500 ms	P0	500 ms	P0	500 ms
H27	1500 ms	F55	1500 ms	H8	1500 ms	H53	1500 ms	F9	1500 ms	H16	1500 ms	F17	1500 ms	F28	1500 ms
P0	500 ms	P0	500 ms	P0	500 ms	P0	500 ms	P0	500 ms	P0	500 ms	P0	500 ms	P0	500 ms
H44	1500 ms	F55	1500 ms	H8	1500 ms	H54	1500 ms	F10	1500 ms	H31	1500 ms	F18	1500 ms	F28	1500 ms
P0	500 ms	P0	500 ms	P0	500 ms	P0	500 ms	P0	500 ms	P0	500 ms	P0	500 ms	P0	500 ms
H44	1500 ms	F67	1500 ms	H4	1500 ms	H54	1500 ms	F79	1500 ms	H20	1500 ms	F48	1500 ms	F31	1500 ms
P0	500 ms	P0	500 ms	P0	500 ms	P0	500 ms	P0	500 ms	P0	500 ms	P0	500 ms	P0	500 ms
H34	1500 ms	F67	1500 ms	H5	1500 ms	H54	1500 ms	F5	1500 ms	H21	1500 ms	F48	1500 ms	F32	1500 ms
P0	500 ms	P0	500 ms	P0	500 ms	P0	500 ms	P0	500 ms	P0	500 ms	P0	500 ms	P0	500 ms
H34	1500 ms	F67	1500 ms	H45	1500 ms	H13	1500 ms	F8	1500 ms	H21	1500 ms	F56	1500 ms	F32	1500 ms
P0	500 ms	P0	500 ms	P0	500 ms	P0	500 ms	P0	500 ms	P0	500 ms	P0	500 ms	P0	500 ms
H34	1500 ms	F34	1500 ms	H45	1500 ms	H15	1500 ms	F66	1500 ms	H21	1500 ms	F21	1500 ms	F76	1500 ms
P0	500 ms	P0	500 ms	P0	500 ms	P0	500 ms	P0	500 ms	P0	500 ms	P0	500 ms	P0	500 ms
H7	1500 ms	F34	1500 ms	H51	1500 ms	H26	1500 ms	F50	1500 ms	H49	1500 ms	F22	1500 ms	F76	1500 ms
P0	500 ms	P0	500 ms	P0	500 ms	P0	500 ms	P0	500 ms	P0	500 ms	P0	500 ms	P0	500 ms
H7	1500 ms	F33	1500 ms	H47	1500 ms	H26	1500 ms	F75	1500 ms	H49	1500 ms	F63	1500 ms	F51	1500 ms
P0	500 ms	P0	500 ms	P0	500 ms	P0	500 ms	P0	500 ms	P0	500 ms	P0	500 ms	P0	500 ms
P0	30.000 ms	P0	30.000 ms	P0	30.000 ms	P0	30.000 ms	P0	30.000 ms	P0	30.000 ms	P0	30.000 ms	Einde eers	30.000 ms

H = House picture; F = Face picture; P0 = Rest
